# Developing genome-wide SNPs and constructing an ultrahigh-density linkage map in oil palm

**DOI:** 10.1038/s41598-017-18613-2

**Published:** 2018-01-12

**Authors:** Bin Bai, Le Wang, Ying Jun Zhang, May Lee, Rahmadsyah Rahmadsyah, Yuzer Alfiko, Bao Qing Ye, Sigit Purwantomo, Antonius Suwanto, Nam-Hai Chua, Gen Hua Yue

**Affiliations:** 1 Temasek Life Sciences Laboratory, 1 Research Link, National University of Singapore, Singapore, 117604 Singapore; 2 R&D Department, Wilmar International Plantation, Palembang, 30118 Indonesia; 3 Biotech Lab, Wilmar International, Cikarang, Bekasi, 17530 Indonesia; 4 Bogor Agricultural University, Bogor, Jawa Barat 16680 Indonesia; 5 Laboratory of Plant Molecular Biology, The Rockefeller University, New York, 10065 USA; 6 Department of Biological Sciences, National University of Singapore, Singapore, 117558 Singapore; 7 School of Biological Sciences, Nanyang Technological University, 6 Nanyang Drive, Singapore, 637551 Singapore

## Abstract

Oil palm (*Elaeis guineensis* Jacq.) is the leading oil-producing crops and the most important edible oil resource worldwide. DNA markers and genetic linkage maps are essential resources for marker-assisted selection to accelerate genetic improvement. We conducted RAD-seq on an Illumina NextSeq500 to discover genome-wide SNPs, and used the SNPs to construct a linkage map for an oil palm (*Tenera*) population derived from a cross between a Deli *Dura* and an AVROS *Pisifera*. The RAD-seq produced 1,076 million single-end reads across the breeding population containing 155 trees. Mining this dataset detected 510,251 loci. After filtering out loci with low accuracy and more than 20% missing data, 11,394 SNPs were retained. Using these SNPs, in combination with 188 anchor SNPs and 123 microsatellites, we constructed a linkage map containing 10,023 markers covering 16 chromosomes. The map length is 2,938.2 cM with an average marker space of 0.29 cM. The large number of SNPs will supply ample choices of DNA markers in analysing the genetic diversity, population structure and evolution of oil palm. This high-density linkage map will contribute to mapping quantitative trait loci (QTL) for important traits, thus accelerating oil palm genetic improvement.

## Introduction

Oil palm (*Elaeis guineensis* Jacq) is a leading oil yielding crop in the world, mainly cultivated in the equatorial tropics of Africa, Southeast Asia and South America. Palm oil currently dominates the global vegetable oil economy, contributing to 33% of the total world oil production and 45% of edible oil worldwide^1^. However, one of the major constraints of oil palm production is slow improvement of oil yield per year^2^. To date, although conventional breeding in combination with improved field management has increased crude palm oil yield from 2.6 tons/ha to 4.0 Tons/ha during the past sixty years, there is still a big yield gap between that and the estimated theoretical potential of 18.2 tons/ha^3^.

To accelerate genetic improvement and to study biological questions in ecology and evolution, polymorphic DNA markers are required. Over the past two decades, DNA markers have been widely used for study of genetics, ecology, evolution and conservation in both model and non-model biology^4^. One important type of DNA markers is microsatellites. They are highly polymorphic and occur over ten-thousand locations in a species’ genome^5^. However, microsatellites exist preferentially in heterochromatic regions, and it is difficult to cover the whole genome evenly. In contrast, single nucleotide polymorphisms (SNPs) are widespread nucleotide variations in genomes, the most abundant type of DNA marker, and easily detected at high-throughput in plant and animal genomes^4^. By taking advantage of the recently developed reference genome sequences for plants and animals, it is easy to develop genome-wide DNA markers with gene information resources for study of genetics and evolution^6^. Furthermore, gene-based SNP markers could themselves be causative SNPs for traits, and have been used for developing transcript maps, QTL analysis, association mapping, and synteny analysis in crops^7^. Nevertheless, few oil palm SNPs have been applied in genetic and genomic research^8–10^.

Genome-wide DNA markers are required to construct high-resolution linkage maps. High-density linkage maps are essential for mapping QTL for marker-assisted selection (MAS) to speed up genetic improvement^11–14^, for the discovery of genomic regions responsible for both commercial and adaptive traits in evolutionary biology^15^, the study of genome evolution^16^ and the chromosomal structure variation^17^, as well as for genome assembly^18,19^. For oil palm, the first linkage map was constructed based on restriction fragment length polymorphism (RFLP) markers^20^. The other linkage maps were made using AFLPs (Amplified fragment length polymorphisms), microsatellites and SNPs markers^8,9,21–23^. However, these maps were unsaturated and most contained only hundreds of markers, largely consisting of microsatellite markers and a few SNPs, which are not sufficient to accurately map QTL for important traits. A high-density genetic map is critically important to accurately map QTL for important traits for MAS, and ultimately to accelerate genetic improvement.

By combining the power of next generation sequencing (NGS) with reduced representation, the discovery, validation and assessment of genome-wide markers in studies have undergone a revolutionary transition over the past ten years with the advent of low-cost and high-throughput restriction-site associated DNA sequencing (RAD-seq) technology^24^. In comparison to traditional marker discovery, RAD-seq is less complicated for constructing highly multiplexed, reduced representation libraries even in species with large genomes^25^. It can simultaneously discover and genotype many (e.g. >2000) SNPs within a few days. This technique has successfully been applied to discover thousands of markers in plants such as wheat and barley^26^, sorghum^27^, rice^28^, switchgrass^29^, sesame^30^, and in insects and fish^31^. While more than ten genetic linkage maps are available for oil palm, only two linkage maps was based on around 1000 SNPs^9,32^.

The aim of our study was to discover many genome-wise SNPs and to construct an ultrahigh-density SNP-based linkage map for the oil palm. These identified genome-wide SNPs will facilitate studies on oil palm ecology and evolution, whereas the ultrahigh-density linkage map will be useful in discovering QTL for important traits for marker-assisted selection to accelerate genetic improvement.

## Results

### Microsatellite marker analysis

A total of 123 out of 300 microsatellite markers selected were informative between the two parental palms, a Deli *Dura* and an AVROS *Pisifera*. Both parental trees were used to produce F_1_ *Tenera* for production in Wilmar International Plantation. All the markers were used to genotype 153 F_1_ palms from the F_1_ production population. Finally, 123 microsatellite markers were mapped into the LGs together with the SNPs. The sequences of the microsatellite markers mapped are listed in Additional file Table S1.

### SNP genotyping using micro-chip assay

A set of 288 SNP markers on 16 chromosomes were used to genotype 153 palms and two parental palms by using the 96.96 Dynamic Array Chip (Fluidigm, Singapore), then 188 SNPs were mapped into the linkage groups (LGs) together with other markers in the palm population. The sequence and alleles of the SNP markers mapped are listed in Additional Table S1. Among the 188 SNPs, 39 SNPs were developed from genes associated with lipid synthesis based on the genome annotation of oil palm^33^.

### SNP discovery and genotyping using RAD-seq

Four RAD-seq libraries were constructed using 96 barcodes and the *Pst*I-HF restriction endonuclease. A total of 1,076 million raw reads were obtained from the sequencing of 155 trees. After filtering out low quality and ambiguous barcodes, there remained 1,050 million reads (97.57% of total raw reads). The retained reads of the two parental palms were 11.73 and 8.89 million reads, respectively (Table 1). From the mapping population, 8 out of 153 progeny individuals were discarded from further analysis due to low coverage of sequencing reads. The average number of raw reads across all retained 145 progeny was 7.10 million.

**Table 1:** Minimum, maximum and average retained reads per palm sample.

	**Number of total reads**	**Number of retained reads**
Minimum	2,234,507	2,104,235
Maximum	21,986,459	21,653,929
Average in population	7,280,180	7,101,950
Parental palm 1	11,919,428	11,730,561
Parental palm 2	9,106,481	8,886,897
Total	1,076,562,923	1,050,400,196

Using the sequence data of the two parental samples, a catalogue containing 510,251 loci was built for SNP discovery. After removing the loci with >20% missing data across progeny, a total of 11,394 informative SNPs were genotyped and used for linkage map construction. Additional Table S1 lists, for each marker in the map, its alleles, flanking sequence, name of contig containing it, and its physical position in the contig.

### Construction of a high-density linkage map

**Figure 1:**
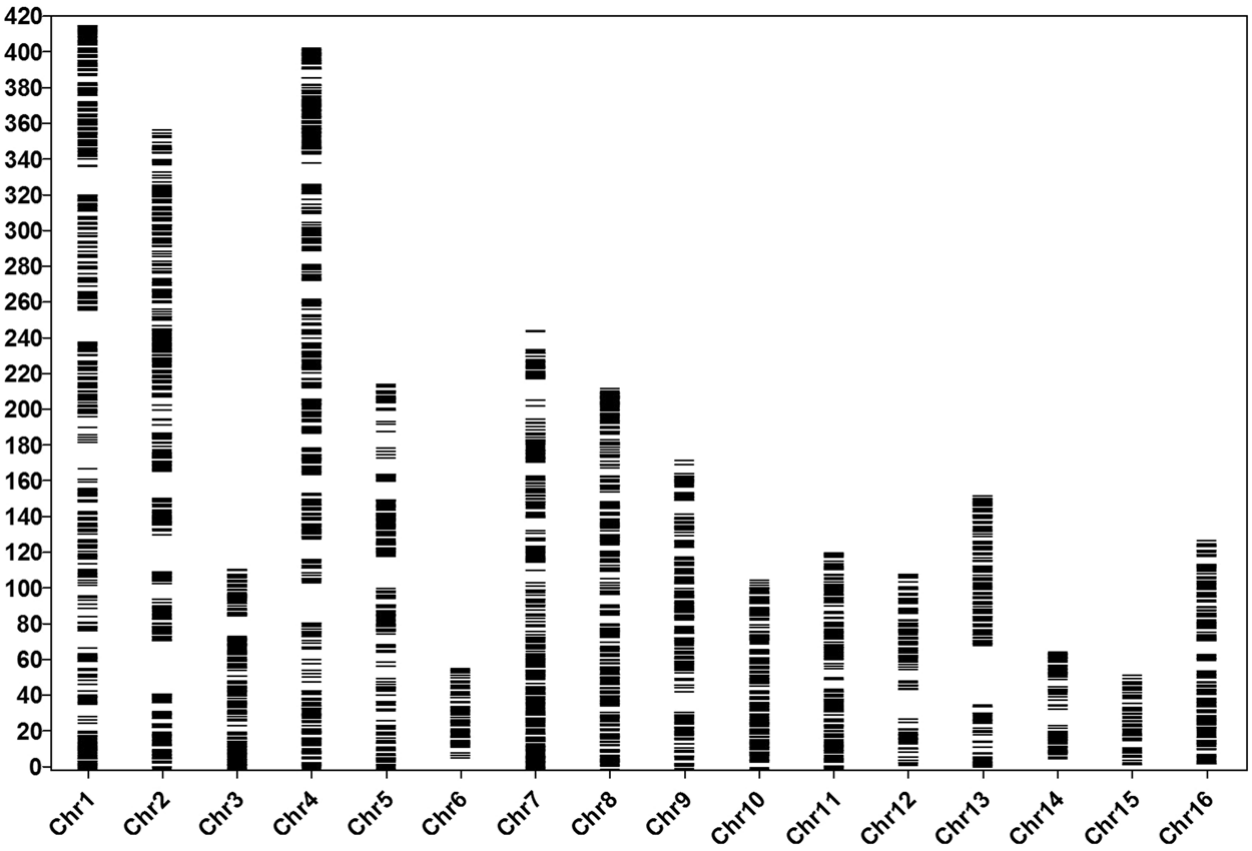
A ultrahigh-density linkage map of African oil palm (*Elaeis guineensis*) with 10,023 markers (i.e. 9900 SNPs markers and 124 microsatellites, see details about the mapped markers in Additional Table S1). The bar on left side represents the map length in cM, whereas the horizontal bars in each linkage group (chromosome) are positions of markers mapped.

The 11,394 SNPs identified from RAD-seq, with another 188 SNPs from micro-chip array and 123 microsatellite markers, were used to construct the linkage map. Of these markers, 1,682 SNPs either could not be assigned to LGs or were assigned to small groups (marker number less than 50), or had segregation distortion (*P* < 0.001), were excluded from further analysis. The retained 10,023 markers were assigned to 16 LGs (Chromosomes) (Fig. 1). The linkage map contained 10,023 markers (9,900 SNPs and 123 microsatellites). The map covered a total length of 2,938.2 cM and had an average marker density of 0.29 cM. The lengths of LGs ranged from 53.47 cM for chromosome (Chr) 15 to 416.78 cM for Chr 1, with an average of 183.64 cM. The marker intervals ranged from 0.17 cM on Chr 10 to 0.46 cM on Chr 13, with an average of 0.29 cM. Marker spaces in all LGs are smaller than 20 cM, except in Chr 2 and Chr 13, with 30.13 cM and 33.39 cM, respectively. A summary of the markers, marker densities and genetic distances for each LG, as well as its corresponding chromosome in the draft genome^1^ are shown in Table 2 and Additional Table S1.

**Table 2:** Summary of the linkage map of African oil palm *Elaeis guineensis* Jacq.

**Chromosome** ^**a**^	**Linkage group** ^**b**^	**Number of markers**	**Length (cM)**	**Marker density (cM)**	**Number of marker per cM**	**Number of gaps (>10 cM)**	**Number of SNPs with gene annotation**
Chr 1	LG 8	1412	416.78	0.3	3.39	3	829
Chr 2	LG 4	1270	358.35	0.28	3.54	3	762
Chr 3	LG 1	597	112.59	0.19	5.3	1	333
Chr 4	LG 11	1214	404.08	0.33	3	5	682
Chr 5	LG 12	532	216.01	0.41	2.46	3	282
Chr 6	LG 10	145	57.08	0.39	2.54	0	67
Chr 7	LG 6	853	245.88	0.29	3.47	1	466
Chr 8	LG 2	872	213.89	0.25	4.08	0	471
Chr 9	LG 7	554	173.53	0.31	3.19	1	344
Chr 10	LG 15	635	106.63	0.17	5.96	0	403
Chr 11	LG 14	525	121.84	0.23	4.31	0	296
Chr 12	LG 13	342	109.63	0.32	3.12	1	196
Chr 13	LG 9	336	153.71	0.46	2.19	1	188
Chr 14	LG 3	183	66.11	0.36	2.77	0	97
Chr 15	LG 16	118	53.47	0.45	2.21	0	54
Chr 16	LG 5	435	128.62	0.3	3.38	0	257
Total		10023	2938.2	0.29	3.41	19	5727

^a^Chromosomes corresponding to those in oil palm genome sequencing (Singh *et al*. 2014); and ^b^Linkage map groups corresponding to those in oil palm published by Billotte *et al*. (2015).

Using the reference genome annotation resource^1^, we searched the genes associated with each SNP. Then 5,727 SNPs were annotated with gene information, with 3,975 SNPs located in intergenic regions. The number of SNPs with gene information in each of the 16 chromosomes ranged from 54 to 829 (Table 2 and Additional Table S1).

## Discussion

After the RAD-seq approach was developed^34,35^, it was initially adopted in a population genomic study^36^. With the continuously decreasing cost of next-generation sequencing (NGS), RAD-seq has become a powerful approach for many research problems, from discovering thousands of SNPs for large-scale population genotyping purposes, to constructing the dense linkage maps for QTL mapping, scaffolding of newly sequenced genomes, and addressing a wide range of questions in molecular ecology and evolutionary biology^37^. Usually, ddRAD-seq with *Pst*I-*Msp*I is used for identification of SNPs in plants and animals^18,38,39^. The first SNP discovery in oil palm using ddRAD-seq with *Pst*I-*Msp*I was conducted with 3417 informative SNPs^9^. In a later study^32^, 2139 informative SNPs were identified using ddRAD-seq with *Pst*I-*Msp*I in an oil palm breeding population, and a high-density SNP genotyping array was developed^40^. In this study, we discovered more than 10,000 informative SNPs using a different approach: single digest RAD-seq with the *Pst*I^35^, modified for a highly heterozygous breeding oil palm population. These indicate that both approaches of RAD-seq could facilitate SNP discovery and improvement of genomic resource. The significant difference in number of SNPs discovered between the two RAD-seq approaches could be caused by restriction enzyme selection, RAD-seq library preparation methods, coverage of sequences reads, as well as the heterozygosity of samples being used in the studies. The previous study showed that variance in read depth among individuals could be minimized, but the expected frequency of restriction cut was decided by a particular restriction enzyme^34^, hence ddRAD-seq with *Pst*I-*Msp*I could result in a lower digest efficiency of endonuclease and hence less common SNPs in the highly heterozygous oil palm samples. The previous study also indicated that the loci discovered across the genome showed the interaction between choice of restriction enzymes and the number of loci recovered from different parts of the genome^41^. Here we provide an evidence that the complexity reduction involving the restriction enzyme *Pst*I-HF was adapted in order to avoid and lower the occurrence of repetitive regions of the high heterozygous oil palm genome. That, in combination with the 300–600 bp size selection for RAD library construction, the library single-end Illumina sequencing by Illumina NextSeq500 in our laboratory, the high throughput reads (150 bp per read) at sufficient depth of coverage which can provide sufficient contigs containing SNPs (Table 1), and reads sequenced with a mean coverage depth of 7~10× which should allow accurately genotyping at most sites across most individuals, means that a simple, highly efficient and lower cost RAD-seq platform for heterozygous oil palm samples has been constructed in this study. The entire process from DNA isolation, library preparation to sequence using Illumina NextSeq500, and SNP calling through *sstacks* pipeline, was relatively simple and fast. Furthermore, the overall cost of RAD-seq was economically efficient. Given the benefit of using this platform in our studies, it may be expected that utilization of this technology will become widely adopted in oil palm and other highly heterozygous samples for SNP discovery, genetic and genomic studies, and this approach has improved the tools developed, alleviating the marker limitation that researchers previously faced for studies of high heterozygous samples.

With the RAD-seq, we discovered over 10 K high-quality and informative SNP markers for oil palm. Among them, 5,727 SNPs are located in genes. These over 10 K SNPs will be useful in studies on ecology, evolution and linkage and QTL mapping for economically important traits to accelerate genetic improvement in oil palm. The genome-wide set of over 5 K SNP markers within genes could facilitate to identity causative SNPs within QTL for important traits, and can be useful in synteny analysis to understand more about genome evolution in plants^7^.

Based on SNPs discovered across the whole oil palm genome, we generated a genome-wide high-density linkage map containing 10,023 markers, distributed through all 16 chromosomes. The length is 2938.2 cM and the average marker density is 0.29 cM. Compared with previous studies, marker numbers in linkage maps have continued to increase, from 252 to 944 microsatellite-based markers^8,21–23^, to 1085 SNP-based markers^9^, and to the SNP-based ultra-high-density linkage map in this study. Also, in terms of marker density, our map is among the highest published to date, compared to the previous marker spacings of 1.26 cM^9^ and 1.4 cM^8^. The high-density linkage map can enable QTL mapping for the gaining of insights into the genome-wide genetic architecture of economic traits, genomic evolutionary studies, genome assembly, revealing the patterns of chromosomal evolution, and linkage disequilibria assessing^4^. Such maps are frequently constructed using microsatellite markers, but microsatellite markers are not as abundant or easily scored as SNPs^4^. However, we noticed that in Chr 2 and 13, in a few positions, marker spaces were bigger than 20 cM. More markers should be mapped in these positions to reduce marker interval for genomic studies. Improvement of the platform of RAD-seq with high efficiency library preparation and bioinformatics procedures used to identify SNPs, as well as the use of large populations for oil palm, could be a feasible solution for this challenge.

In conclusion, we identified many SNPs using RAD-seq, supplying sufficient DNA markers for studying ecology and evolution of oil palm. Then, we constructed an ultra-high-density linkage map with 10,023 markers for African oil palm. To the best of our knowledge, this is the densest linkage map in oil palm. The linkage map will facilitate mapping QTL for economically important traits for MAS to accelerate genetic improvement in oil palm.

## Materials and Methods

### The palm population used for linkage mapping

A F_1_ population was generated by crossing a Deli *Dura* (mother palm) and an AVROS *Pisifera* (father palm) in Wilmar International Plantation. Both parental trees were used to produce *Tenera* trees for production in Wilmar International Plantation. A total of 153 F_1_ progenies (*Tenera* type) were planted into a plantation field in Indonesia in 2006. These F_1_ trees were managed under the same conditions following the standard protocol of Wilmar Plantation in Indonesia (http://www.wilmar-international.com/our-business/tropical-oils/plantations/).

### DNA extraction from palm leaves

DNA was isolated from young leaf samples using DNAeasy Plant Mini Kit (Qiagen, Hilden, Germany) following the manufacturer’s instruction. RNA was removed by RNAse digestion during lysis, and cell debris, precipitated proteins, and polysaccharides were removed by centrifugation through a QIAshredder spin column. Then, the lysate was loaded into the DNEasy Plant Mini spin column, in which DNA selectively binds to the silica membrane. Finally, the remaining contaminants and enzyme inhibitors were removed in one or two efficient wash steps and pure DNA was then eluted in 1xTE buffer. The quality of DNA was checked on 1.0% agarose gels. DNA quantification was conducted using Nanodrop 2000 (Nanodrop, Wilmington, DE, USA). For RAD-seq library construction, the concentration of double-strand DNA was also measured using Quanti-Qubit® dsDNA HS Assay Kit (Thermo Fisher Scientific, Carlsbad, CA, USA) on a microplate reader of Tecan Infinite M200 (Tecan, Männedorf, Switzerland).

### Genotyping of microsatellites

The microsatellites used in this study (see Additional Table S1) were developed by us^22^ using a method described previously^42^ Primers of each microsatellite were designed in the repeat-flanking regions using PrimerSelect (DNAstar, CA, USA). To genotype the microsartellites on the automatic DNA sequencer ABI3730xl (Applied Biosystems, Foster City, CA, USA) one primer for each marker was labelled with a fluorescent dye (i.e. either HEX or 6-FAM). The microsatellite markers were genotyped individually with PCR and electrophoresis for polymorphism and informativeness between the two parental trees. Each microsatellite marker was amplified using PCR, which contained 40 ng of genomic DNA, 2.0 units of DNA Taq-DNA-polymerase (Bio-Rad, Berkeley, CA, USA), 1× PCR buffer containing 1.5 mM MgCl_2_, 0.2 µM dNTPs and 50 nM of each primer. PCR was carried out on PTC100-PCR machines (Bio-Rad, Berkeley, CA, USA). The following PCR conditions were applied: three minutes of denaturation at 94 °C, 35 cycles of 30 s at 94 °C, 30 s at 53–58 °C and 45 s at 72 °C; and a final extension at 72 °C for 10 min. The PCR products were electrophoresed and analysed on an ABI3730x1 sequencer (Applied Biosystems, Foster City, CA, USA). The allele sizes and genotypes were analysed against the ROX-500 standard using GeneMapper 4.1 (Applied Biosystems, Foster City, CA, USA). The genotype data were exported for later linkage mapping.

### Genotyping of SNPs using micro-chip based on allele-specific competitive PCR assay

From over 10,000 SNPs identified in the 17 re-sequenced palm genomes^33^, 288 SNPs were randomly selected from 16 chromosomes as anchor markers based on their genomic position from previously published genome^1^. The SNPs were genotyped using allele-specific competitive PCR assay according to our method^43^, which is a homogenous, fluorescence-based genotyping technology that is based on allele-specific oligo extension and fluorescence resonance energy transfer for signal generation. The SNP assay consisted of three parts. Firstly, the primer design: the SNP allele-specific forward primers and a common reverse primer were designed based on an approximately 120 bp long genomic sequence flanking each SNP site (http://bioinfo.biotec.or.th/WASP/). The two probes with fluoresce-nt labels (FAM and HEX, respectively) complement the tail sequence of the allele-specific forward primers. Secondly, loading the assay components: DNA sample (50 ng/µl), 2× Biotium Fast Probe qPCR Master Mix buffer, forward primer mix (0.25 µM), reverse primer (0.25 µM), probe primers mix (10 µM), ROX (50×), 20× sample loading reagent, and two probe primers were mixed together with the DNA sample and then loaded into the sample cell, and the two forward primers and the reverse primer were mixed together and loaded into primer cell in the 96.96 Dynamic Array^TM^ IFC (Fluidigm, South San Francisco, CA, USA). Finally, the PCR reaction: The reaction was performed in a Biomark HD (Fluidigm, South San Francisco, CA, UAS) with following steps: 94 °C for 15 min; followed by 9 cycles of 94 °C for 20 s; and annealing at 65 °C (−1 °C per cycle) for 20 s, and 72 °C for 20 s; then 30 cycles of 94 °C for 10 s, 57 °C for 1 min and 72 °C for 20 s; and a final extension of 72 °C for 3 min. Then the SNP genotypes of the samples were analysed based on the two parental samples’ alleles.

### Construction of RAD-seq libraries and sequencing

The RAD-seq libraries were constructed according to the published method^35^ with some modifications. A total of 153 trees of the breeding population and two parental trees were used for library construction. Briefly, 500 ng of genomic DNA from each individual was digested with high-fidelity *Pst*I (New England Biolabs, Ipswich, MA, USA), and the digested fragments were then ligated to barcoded adapters. The ligation products with unique barcodes were pooled and sheared with Covaris M220 (Covaris, Woburn, MA USA) with a DNA fragment peak of 500 bp. The sheared DNA was purified with MinElute PCR purification kit (Qiagen, Hilden, Germany) and then size selected (300–600 bp) with Agencourt AMPure XP beads (Beckman coulter life sciences, Brea, CA, USA). Recovered DNA was blunt end repaired using Quick Blunting Kit (New England Biolabs, Ipswich, MA) and then A-overhangs were added with klenow fragment (New England Biolabs, Ipswich, MA, USA). Sequencing adaptor P_2_ was then ligated to the DNA fragments with T4 DNA ligase (New England Biolabs, Ipswich, MA, USA). Finally, the library was amplified using Phusion® High-Fidelity DNA Polymerase (New England Biolabs, Ipswich, MA, USA) and the PCR products were purified using Agencourt AMPure XP beads. The recovered library was quantified with Illumina library quantification kit (Kapa Biosystems, Wilmington, MA, USA) in the MyiQ Thermal Cycler (Bio-Rad, Hercules, CA, USA) and sequenced for 1 × 150 bp reads on NextSeq500 (Illumina, San Diego, CA, USA).

### Identifying of SNP from RAD-seq and genotyping

The raw sequencing reads were demultiplexed and cleaned using the program *process*_*radtags* in the software package Stacks v1.42^34^. Any uncalled base and low quality reads were removed. To minimize the sequencing errors, all the clean reads were trimmed to 130 bp. In detail, only the reads with a limit of >Q30 for Illumina sequencing were used for further analysis, the SE reads were then trimmed to 130 bp from the 3′ end and that of any uncalled base were removed. The reads with an average score within sliding window of <10 were also removed. The cleaned reads were aligned to the oil palm reference genome^1^ by the use of the program GSNAP with a maximum mismatch of 5 bp. Only the alignments of unique targets were used for stacks assembly using the program *pstacks* (−m, 3). The assembled stacks from the parents were used to build a catalogue of loci using the program *cstacks* in the Stacks package. The assembled stacks from each sample were then matched to the catalogue for SNP discovery using *sstacks*. Finally, the program *genotypes* was used to call SNPs across progeny. The genotyped SNPs were filtered with a cut-off of at least 10 × sequence depth and at most 20% missing data. Then the genes associated with SNP loci were searched against the oil palm genome annotation^1^. Markers heterozygous in any one parent or both parents were selected for further analysis. There are five different allelic patterns in all called markers: type *nn* × *np* (segregating 1:1) was homozygous in the male parent and heterozygous in the female parent; *lm* × *ll* (1:1) was heterozygous in the male parent and homozygous in the female parent; *hk* × *hk* (1:2:1) was heterozygous in both parents with two shared alleles; *ef* × *eg* (1:1:1:1) was heterozygous in both parents with two sex-specific alleles and one shared allele, and type ab × cd (1:1:1:1) was heterozygous in both parents with four different alleles.

### Linkage map construction

Linkage analysis was performed for markers genotyped in F_1_ palms derived from crossing *Dura* and *Pisifera*. Polymorphic markers from microsatellites and anchor SNPs, and SNPs from RAD-seq, were used to construct the linkage map. The genetic linkage map was generated using software Lep-MAP2^44,45^. Before linkage map analysis, the default dataTolerance value of 0.001 (χ^2^ test, *P* < 0.001) was used to filter out markers with segregation distortion. Then the remaining loci were grouped into linkage groups (LGs) using a LOD score threshold of 10. The order and positions of the markers within their respective LGs were determined according to the recombination probabilities between loci, with the sex-averaged option selected. And the genetic maps were drawn using the software MapChart V2.2^46^. Finally, the LGs for the mapping population were numbered according to the published linkage map (Billotte *et al*.^21^) based on the anchor markers and genomic sequence^1,21^.

### Data availability

The RAD-seq datasets supporting the conclusions of this article are available in the DDBJ database, accession number (BioProject Accession: PRJDB5817).

## Electronic supplementary material

Table S1
